# Population-based study on surgical care for primary spontaneous pneumothorax

**DOI:** 10.1093/ejcts/ezae104

**Published:** 2024-03-15

**Authors:** Quirine C A van Steenwijk, Louisa N Spaans, David J Heineman, Frank J C van den Broek, Chris Dickhoff

**Affiliations:** Department of Surgery, Maxima Medical Centre, Veldhoven, Netherlands; Department of Cardiothoracic Surgery, Amsterdam UMC, Vrije Universiteit Amsterdam, Amsterdam, Netherlands; Department of Surgery, Maxima Medical Centre, Veldhoven, Netherlands; Department of Cardiothoracic Surgery, Amsterdam UMC, Vrije Universiteit Amsterdam, Amsterdam, Netherlands; Department of Surgery, Maxima Medical Centre, Veldhoven, Netherlands; Department of Cardiothoracic Surgery, Amsterdam UMC, Vrije Universiteit Amsterdam, Amsterdam, Netherlands

**Keywords:** Primary spontaneous pneumothorax, Surgical technique, Postoperative complications, Length of stay

## Abstract

**OBJECTIVES:**

The optimal surgical strategy for primary spontaneous pneumothorax remains a matter of debate and variation in surgical practice is expected. This variation may influence clinical outcomes, such as postoperative complications and length of stay. This national population-based registry study provides an overview and extent of variability of current surgical practice and outcomes in the Netherlands.

**METHODS:**

To identify national patterns of care and between-hospital variability in the treatment of primary spontaneous pneumothorax, patients who underwent surgical pleurodesis and/or bullectomy between 2014 and 2021, were identified from the Dutch Lung Cancer Audit—Surgery database. The type of surgical intervention, postoperative complications, length of stay and ipsilateral recurrences were recorded.

**RESULTS AND CONCLUSIONS:**

Out of 4338 patients, 1851 patients were identified to have primary spontaneous pneumothorax. The median age was 25 years (interquartile range 20–31) and 82% was male. The most performed surgical procedure was bullectomy with pleurodesis (83%). The overall complication rate was 12% (Clavien–Dindo grade ≥III 6%), with the highest recorded incidence for persistent air leak >5 days (5%). Median postoperative length of stay was 4 days (interquartile range 3–6) and 0.7% underwent a repeat pleurodesis for ipsilateral recurrence. Complication rate and length of stay differed considerably between hospitals. There were no differences between the surgical procedures. In the Netherlands, surgical patients with primary spontaneous pneumothorax are preferably treated with bullectomy plus pleurodesis. Postoperative complications and length of stay vary widely and are considerable in this young patient group. This may be reduced by optimization of surgical care.

**Figure:**
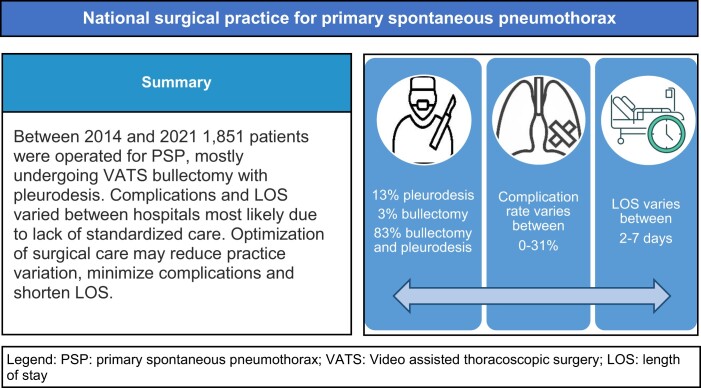

## INTRODUCTION

Spontaneous pneumothorax is a clinical condition with an estimated incidence of 22.7 per 100 000 patients per year [1]. In general, spontaneous pneumothorax is divided into primary spontaneous pneumothorax (PSP) or secondary spontaneous pneumothorax (SSP). PSP is mostly diagnosed in young and healthy males with an incidence of 12.3 cases per 100 000 males [2].

The treatment of PSP is primarily conservative using aspiration or chest tube drainage, irrespective of the size of pneumothorax [3, 4]. Moreover, in patients without significant clinical symptoms, an observational policy is also shown to be effective in resolving moderate-to-large PSP within 8 weeks [5]. However, after conservative treatment with a chest tube, recurrence rates up to 48% are reported [2]. In case of recurrent ipsilateral spontaneous pneumothorax or persistent air leak (PAL) at first presentation, surgical pleurodesis is recommended in guidelines [5, 6]. This is already standard of care in the Netherlands. However, there is no consensus on the optimal surgical strategy, e.g. chemical or mechanical pleurodesis and the role of apical wedge resection or bullectomy [7, 8]. As results from low-quality studies investigating the optimal surgical approach for PSP are contradictive and inconsistent, solid recommendations for the best treatment of PSP are lacking. This potentially results in considerable variability in surgical care, complication rate, length of hospital stay (LOS) and patient satisfaction [9]. Besides, surgical treatment-related data on LOS, morbidity and mortality and their subsequent impact on the health care system is lacking.

In the Netherlands, all benign or malignant thoracic surgical procedures are compulsorily registered in a nationwide Dutch Lung Cancer Audit for Surgery (DLCA-S) database for quality improvement. The aim of this study is to get insight in the extent of surgical variability in the Netherlands regarding treatment of PSP and in particular its impact on postoperative outcomes. This study may expose relevant variability, which can be subject for future studies aiming to optimize and standardize surgical care for PSP.

## PATIENTS AND METHODS

### Source

Patients surgically treated for pneumothorax were identified from the DLCA-S database, after approval by the privacy review board. The DLCA-S is one of the registries of the Dutch Institute for Clinical Auditing (DICA), which was founded in 2010 to register patterns of care for a variety of clinical conditions. The DLCA-S was established in 2012 as a national audit to monitor and evaluate the quality of pulmonary surgery in the Netherlands. In the first years, patient registration was voluntary, but since 2015, it is mandatory, resulting in a comprehensive registration of all lung surgical procedures in the Netherlands [6]. In accordance with the regulations of the Central Committee on Research involving Human Subjects (CCMO), approval from an ethics committee is not required in the Netherlands.

### Study design and patient selection

For this study, all patients who were registered for the terms ‘bullectomy’, ‘pleurodesis’ or ‘bullectomy combined with pleurodesis’ during the period 1 January 2014 until 1 January 2021, were selected. However, pleurodesis is not further specified in the DLCA-S, resulting in an aggregation of complete or partial surgical pleurectomy, pleural abrasion and chemical pleurodesis (e.g. talcage). Patients who had an additional surgical procedure, such as chest wall resection or diaphragm surgery, and those who had a registered history of ipsilateral thoracic surgery and/or radiotherapy were excluded. As the DLCA-S database does not register long-term follow-up, no accurate data on recurrence of pneumothorax could be established. Duplicate cases (i.e. patients who underwent pleurodesis twice during the investigated period), however, were used to assess repeat pleurodesis for recurrent pneumothorax after pleurodesis. Only the repeat procedures were excluded for the primary analysis. Since the distinction between PSP and SSP is not recorded in the DLCA-S database as separate diagnoses, we counselled the available literature to select a subgroup of patients with a very high probability to be treated for PSP, and not SSP: patients aged between 16 and 39 years, having an American Society of Anesthesiologists (ASA) score of 1–2 and with Eastern Cooperative Oncology Group (ECOG) performance status of 0–1 [1].

### Outcome measures

The primary outcome was to assess the frequency of surgical procedures, postoperative complications (<30 days) and LOS for patients treated for PSP. Secondary outcomes were incidence of repeat pleurodesis, and assessment of variability in complications and LOS between hospitals regarding the treatment of PSP.

The following variables were extracted from the registry: age, gender, ASA, ECOG performance status, type of surgery (bullectomy, pleurodesis and bullectomy plus pleurodesis), surgical approach (video-assisted thoracoscopic surgery (VATS) and thoracotomy), perioperative blood loss, postoperative complications, LOS and repeat pleurodesis (duplicates in the database). Postoperative complications were rated based on the Clavien–Dindo classification system [7]. PAL was defined as persistent air leak for more than 5 days.

### Statistical analysis

Results were analysed with descriptive statistics using Statistical Package for Social Sciences (SPSS, version 22.0, IBM, Armonk, NY) according a pre-specified statistical analysis plan. Categorical baseline characteristics of all included patients were compared using the chi-square test. Continuous variables were analysed using the independent *t*-test or one-way ANOVA test in case of normal distributed data. In case data were not normally distributed, the Mann–Whitney *U* or Kruskal-Wallis test was used. The Kolmogorov–Smirnov test was used to assess the distribution of the data. Continuous variables were expressed as mean with standard deviation in case of a normal distribution and otherwise with median and interquartile range. Categorical variables were expressed as number with percentages (%). *P* < 0.05 was considered statistically significant.

## RESULTS

**Figure 1:**
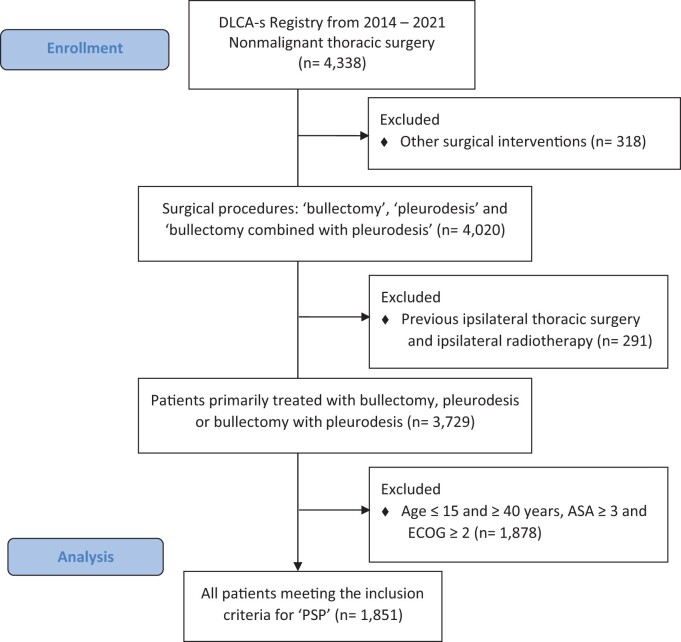
CONSORT 2010 flow diagram. Patient selection with PSP from the DLCA-s registry. ASA: American Society of Anesthesiologists; DLCA-s: Dutch Lung Cancer Audit for Surgery; ECOG: Eastern Cooperative Oncology Group; PSP: Primary Spontaneous Pneumothorax.

Figure 1 shows the flowchart of the patient selection from the DLCA-S registry. From 2014 to 2021, 4338 surgical procedures for pneumothorax were registered in the DLCA-S. Exclusion of patients who underwent additional thoracic surgical procedures or previous ipsilateral surgery or radiotherapy resulted in 3729 eligible patients. Of these, 1851 patients fulfilled the before-mentioned inclusion criteria.

### Primary spontaneous pneumothorax

For the 1851 patients fulfilling our criteria of PSP, median age was 25 (interquartile range 20–31) years and 82.2% was male. Results of the 3 different surgical techniques are presented in Table 1, with bullectomy plus pleurodesis being the most performed surgical procedure (83.3%). Postoperative complications <30 days occurred in 226 patients (12.2%; 95% CI 10.8–13.8), without significant differences between the 3 surgical techniques. Complications were PAL, postoperative haemorrhage, infection (e.g. pneumonia, wound infection) and other (not further specified). In some patients, more than 1 complication occurred. The median LOS was 4.0 (interquartile range 3.0–6.0) days and did not differ between surgical techniques.

**Table 1:** Patient characteristics of the defined PSP subgroup

		Total	Pleurodesis	Bullectomy	Bullectomy + pleurodesis	*P*-value
Patients^a^, *n* (%)		1851 (100)	246 (13.2)	63 (3.4)	1542 (83.3)	
Surgical approach, *n* (%)	VATS	1809 (97.7)	240 (97.6)	62 (98.4)	1507 (97.7)	0.575^b^
	Thoracotomy	8 (0.4)	2 (0.8)	1 (1.6)	5 (0.3)	
	Conversion	2 (0.1)	0 (0.0)	0 (0.0)	2 (1.7)	
	Other^c^	32 (1.7)	4 (1.6)	0 (0.0)	28 (1.8)	
Gender, *n* (%)	Male	1520 (82.2)	193 (78.5)	49 (77.8)	1278 (82.9)	0.153^b^
	Female	330 (17.8)	53 (21.5)	14 (22.2)	263 (17.1)	
Age, *n* (%)	Median (IQR)	25 (20.0–31.0)	23 (20.0–30.0)	27 (20.0–33.0)	25 (20.0–31.0)	0.230^d^
Complications <30 days, *n* (%)		226 (12.2)	37 (15.0)	8 (12.7)	181 (11.7)	0.337^b^
Clavien–Dindo, *n* (%)						
1–2		117 (6.3)	21 (8.5)	2 (3.2)	94 (6.1)	0.537^b^
3		94 (5.1)	13 (5.3)	5 (7.9)	76 (4.9)	
4		15 (0.8)	3 (1.2)	1 (1.6)	11 (0.7)	
5		0 (0.0)	0 (0.0)	0 (0.0)	0 (0.0)	
Type of complications, *n* (%)						
PAL >5 days		89 (4.8)	16 (6.5)	4 (6.3)	69 (4.5)	0.325^b^
Postoperative haemothorax		34 (1.8)	4 (1.6)	2 (3.2)	28 (1.8)	0.708^b^
Postoperative infection		27 (1.5)	4 (1.6)	0 (0.0)	23 (1.5)	0.609^b^
Other^e^		86 (4.6)	16 (6.5)	1 (1.6)	69 (4.5)	0.187^b^
Reintervention, *n* (%)		107 (5.8)	16 (6.5)	6 (9.5)	85 (5.5)	0.357^b^
LOS (days), median (IQR)		4.0 (3.0–6.0)	4 (3.0–6.0)	4 (3.0–6.0)	4 (3.0–6.0)	0.223^d^

IQR: interquartile range; LOS: length of stay; PAL: persistent air leak >5 days; VATS: video-assisted thoracoscopic surgery.

a Sum of percentages 100% in row for number of patients.

b Chi-square test was used.

c Approach not further described.

d Kruskal–Wallis test was used. Not all data were complete per characteristic, missing values are not presented in the table and hence denominators can slightly differ.

e Other complications included chylothorax (*n* = 1), atelectasis (*n* = 2), recurrent laryngeal nerve damage (*n* = 1), bronchopleural fistula (*n* = 1), air leak not further specified (*n* = 49), complications not further specified (*n* = 32).

### Repeat pleurodesis after surgery for primary spontaneous pneumothorax

In the study period, 110 (5.9%) duplicate cases were identified of which 13 (0.7%) with ipsilateral recurrence, 96 (5.2%) with contralateral recurrence and 1 (0.05%) case in which the side of surgery was unknown. The median time to 2nd surgery was 64 days (CI 34.5–407.5; range 10–924 days).

### Outcomes primary spontaneous pneumothorax per hospital

**Figure 2:**
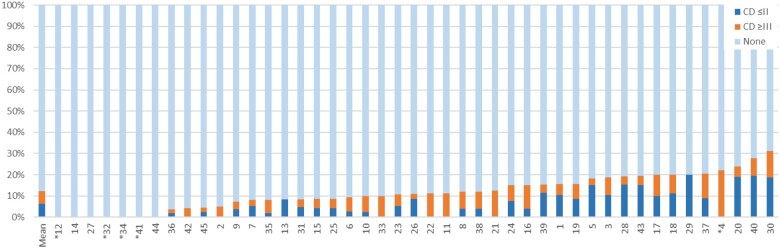
Percentage of complications within 30 postoperative days per hospital in the PSP subgroup (*n* = 1851 patients with PSP; period 2014–2021). CD: Clavien–Dindo; PSP: Primary Spontaneous Pneumothorax. *Hospitals with <10 patients operated.

**Figure 3:**
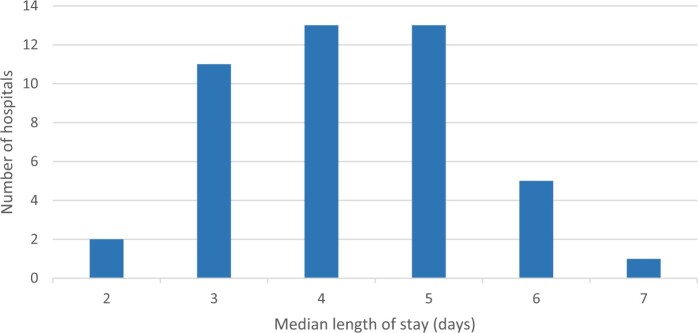
Distribution of median postoperative length of stay in days per hospital for patients treated for PSP [*n* = 1832 patients with PSP (19 missing); period 2014–2021]. PSP: Primary Spontaneous Pneumothorax.

Between 2014 and 2021, surgery for PSP was performed in 45 hospitals with a varying number of 1–107 surgeries per hospital. Bullectomy plus pleurodesis was the preferred surgical intervention in most hospitals. Complication rates per hospital are shown in Fig. 2 and varied between hospitals with a range from 0 to 31% (*P* < 0.001; Chi-square test). Likewise, the median LOS of patients treated for PSP varied between hospitals from 2 to 7 days (*P* < 0.001; Kruskal–Wallis test), see Fig. 3.

## DISCUSSION

This prospective national database study describes both the variability in types of surgical treatment used for PSP, as well as the variation of surgical outcomes among hospitals. VATS bullectomy combined with pleurodesis was found to be the most performed surgical procedure (83%). Although surgery in young healthy patients with PSP is not considered complex, postoperative complications occurred in 12% (Clavien–Dindo grade ≥III in 6%) and the complication rates varied between hospitals from 0 to 31%. Moreover, median LOS was 4 days, which varied between hospitals from 2 to 7 days. The goal of the DLCA-S registry is to identify best practices and negative outliers in order to optimize outcomes nationwide. Optimizing surgical care of PSP may reduce treatment variation, minimize postoperative complications, shorten LOS and hence may also reduce health care costs [8].

The preference of surgical bullectomy and pleurodesis in PSP is in accordance with current guidelines, which recommend bullectomy as the 1st surgical step, followed by pleurodesis to prevent recurrent pneumothorax [3, 10]. This recommendation is based on the hypothesis that the rupture of bullae is the underlying cause of PSP, and therefore bullectomy or apical wedge resection is generally accepted as standard surgical procedure. However, bullectomy was part of surgery in only 86% (1373/1597) of our cases. A possible explanation for omitting bullectomy might be the absence of visible bullae during surgery or preoperative imaging. Performing routine apical wedge resection in these patients remains a matter of debate as well. Czerny *et al.* showed that pleurectomy with apical wedge resection in patients without bullae did result in a significant reduction of recurrences compared to pleurectomy alone [11]. On the other hand, regeneration of bullae near the staple line after bullectomy or standard apical wedge resection is also postulated to be an important cause for recurrence, suggesting that standard apical wedge resection in patients without visible bullae should not be performed and resection of lung parenchyma should be limited [12–14]. An ongoing randomized controlled trial currently investigates whether additional wedge resection is superior compared to pleurectomy alone [15]. The results of this trial are awaited and will clarify the abovementioned debate.

The 2nd surgical step is to perform pleurodesis [3, 10]. Pleurodesis can be achieved mechanically, e.g. by pleurectomy or pleural abrasion, and chemically by using talc. Pleurodesis was done in 97% (1788/1851) of our PSP patients, but unfortunately the DLCA-S registry does not record type of pleurodesis. However, a recent survey regarding surgical treatment for PSP among Dutch thoracic surgeons showed that 80% perform pleurectomy and 20% talcage [9]. As this survey included 70% of all Dutch thoracic hospitals, these data reliably represent the used pleurodesis technique in the Netherlands. A systematic review and meta-analysis about the optimal surgical technique for pleurodesis concluded that bullectomy with additional chemical pleurodesis results in the lowest recurrence rates compared to other surgical techniques [16]. Another more recent systematic review and meta-analysis compared chemical and mechanical pleurodesis, demonstrating lower recurrence rates for chemical pleurodesis as well [17]. In both reviews, pleural abrasion as well as pleurectomy were both analysed as mechanical pleurodesis, which among other factors resulted in high heterogeneity and predominantly low methodological quality. Therefore, firm recommendations on preferred surgical technique cannot be made, and high-quality research comparing mechanical and chemical pleurodesis is necessary.

While the debate on best surgical technique is ongoing, between-hospital variation was observed in complication rates ranging from 0 to 31%. Next to the used surgical technique factors of perioperative care (e.g. chest tube and pain management), level of experience and completeness of complication registration may all clarify this variability. Despite missing a clear clarification, this wide variation in complication rate is a divulgence that should be addressed. Interestingly, equal postoperative complication rates were found for pleurodesis alone, bullectomy alone and the combination of bullectomy and pleurodesis (*P* = 0.337). The most frequent complication was PAL with an incidence of 5%. In case ‘air leak not further specified’ was registered as PAL as well, this complication was reported in up to 7.5% (Table 1), making it an important factor for prolonged LOS and possibly a risk factor for postoperative recurrence [18]. Our results are consistent with the literature where an incidence up to 8% is reported [19]. Incomplete bullectomy, unsuccessful pleurodesis, parenchymal damage or staple-line failure might be causes for PAL. Complete bullectomy and pleurodesis together might prevent the occurrence of PAL, which is also suggested by our data as the combination had the lowest incidence of PAL (4.5%) compared to bullectomy (6.3%) or pleurodesis (6.5%) alone.

Next to variability in complications, we also demonstrated large between-hospital variation in postoperative LOS ranging from 2 to 7 days. This variation can be partly explained by the differences in complications, but also by differences in perioperative care (e.g. chest tube and pain management). A previous study using DLCA-S data to assess the postoperative LOS after minimally invasive lung resection also demonstrated such between-hospital variation in postoperative LOS [20]. This variation was most likely attributed to differences in perioperative care, e.g. adhering to the enhanced recovery after thoracic surgery (ERATS) protocol. Although the median postoperative LOS in our study was 4 days, which is in line with others reporting a LOS of 3–7 days [16, 17], some progressive researchers who focused on the safety and feasibility of early postoperative chest tube removal reported a mean postoperative LOS of only 1 day in patients operated for PSP [21, 22]. Since chest tube duration is not registered in the DLCA-S, it remains unclear whether LOS in our population is determined by extensive chest tube duration protocols. High-quality evidence is necessary to make firm recommendations about optimal chest tube management, as it has considerable impact on chest tube duration and LOS [19]. Likewise, postoperative analgesia also may influence complications and LOS, as well as patient satisfaction. The use of loco-regional analgesia is currently recommended over thoracic epidural analgesia since they lack the epidural related side-effects (e.g. hypotension, urinary retention and muscle weakness) [23]. This enables early mobilization, possibly decreasing complications and reducing LOS. A recent survey among Dutch thoracic surgeons showed that after pleurodesis thoracic epidural analgesia still was the preferred analgesic technique in 78% of respondents [9]. Based on our demonstrated variation in complications and LOS among patients with PSP, a nationwide randomized trial (ClinicalTrials.gov Identifier: NCT06053476) in PSP patients has started in order to evaluate the role of chest tube and pain management on recovery.

When optimizing perioperative care regarding complications and LOS, recurrence rates should also be taken into consideration. Our results demonstrated that 0.7% of the patients operated for PSP needed a repeat pleurodesis for ipsilateral recurrence in the evaluated period from 2014 to 2021. Little is known about the amount of patients requiring repeat surgery for recurrence. Only 2 old studies from more than 15 years ago described percentages of 2–3% requiring repeat VATS/thoracotomy for recurrence [24, 25]. The total number of ipsilateral recurrences after VATS in our population will be higher than 0.7% since some patients will be treated with chest tube drainage or observation alone, which is not registered in our surgical database.

Although all Dutch hospitals are obliged to register their lung surgical data in the DLCA-S and therewith this study represents comprehensive population-based data, the registry has limitations. Since DLCA-S data are published transparently for auditing purposes, registration should preferably be done by independent data- and monitoring specialists. However, the DLCA-S registry allows surgeons to complete their data themselves, which may create ‘publication’ bias. Despite registration being mandatory, data were not always complete. Regarding our primary outcome measures, however, only LOS had missing values. The percentage of missing values amounted to 1.0% in the PSP group, which has no significant influence on the reliability of our results due to the large numbers. Data of the DLCA-S registry has been validated by external parties and shown to be reliable and generalizable [26]. Mainly because of the limited number of registered variables, this study did not include some important factors, such as surgical indication, chest tube duration and analgesia. The registry also lacks follow-up data to provide exact recurrence rates; however, reporting repeat pleurodesis for ipsilateral recurrence was possible. Moreover, while we postulate that adhering to standardized care protocols may lead to a reduction in healthcare expenses, this study did not cover an assessment of costs or resource utilization. Additionally, from a societal perspective, the return to work outcomes could conceivably be affected by standardized care, fewer complications and a shorter LOS.

## CONCLUSION

This study provides a concise overview of current surgical practice for PSP in the Netherlands and between-hospital differences in outcomes. The preferred surgical technique is pleurodesis combined with bullectomy or apical wedge resection. Pleurodesis, bullectomy and bullectomy with pleurodesis have comparable complication rates and LOS. However, there is considerable and relevant variation in outcomes between hospitals. Therefore, we conclude that there is a knowledge gap for a frequently encountered disease that should be the topic of future studies. High-quality research, also considering healthcare costs and resource utilization, is necessary to optimize and standardize perioperative care to improve complication rate and shorten LOS.

## Data Availability

The data underlying this article will be shared on reasonable request to the corresponding author.

## Notes

Presented at the ESTS Annual Meeting 2023, Milan

## ABBREVIATIONS

ASA: American Society of Anesthesiologists
CCMO: Central Committee on Research involving Human Subjects
DICA: Dutch Institute for Clinical Auditing
DLCA-S: Dutch Lung Cancer Audit for Surgery
ECOG: Eastern Cooperative Oncology Group
ERATS: Enhanced recovery after thoracic surgery
LOS: Length of hospital stay
PAL: Persistent air leak
PSP: Primary spontaneous pneumothorax
VATS: Video-assisted thoracoscopic surgery
